# The Association of Volunteer Motivation and Thriving at Work of College Students During COVID-19: Job Burnout and Psychological Capital as Mediators

**DOI:** 10.3389/fpubh.2022.923196

**Published:** 2022-06-22

**Authors:** Jun Li, Cao Ge, Shiyi Li

**Affiliations:** ^1^Faculty of Psychology, Tianjin Normal University, Tianjin, China; ^2^College of Education, Zhengzhou University, Zhengzhou, China; ^3^Tianjin Normal University, Academy of Psychology and Behavior, Tianjin, China; ^4^Tianjin Social Science Laboratory of Students' Mental Development and Learning, Tianjin, China

**Keywords:** volunteer motivation, thriving at work, job burnout, psychological capital, college student volunteer, COVID-19

## Abstract

Thriving at work is a type of mental state in which an individual feels vigorous and learning at the same time in the job. Previous studies have shown that individual internal motivation is relevant to thriving at work and volunteer behaviors, but the role of motivation is still to be further explored. Based self-determination theory, this study focuses on the mediating effects of job burnout and psychological capital on the relationship between volunteer motivation and thriving at work. Three hundred forty-nine college student volunteers who participated in psychological assistance volunteer activities during the COVID-19 pandemic were investigated using the Volunteer Function Motivation Inventory, Maslach Burnout Inventory, PsyCap Questionnaire, and Thriving at work scale. The results indicated that job burnout and psychological capital mediate the relationship between volunteer motivation and thriving at work. The results not only offer important theoretical insights of Volunteer Motivation and Thriving at Work, but also generate practical implications regarding how to use motivating Volunteer behavior and enhanced wellbeing at work.

## Introduction

During the COVID-19 pandemic in 2020, many college volunteers participated in pandemic prevention and control activities and performed various types of volunteering work in China (1). Volunteering is a long-term and free act of helping those who actively seek help after careful consideration in the context of an organization (2). Volunteering during the COVID-19 pandemic's prevention and control period has certain characteristics, and every volunteer is not only a participant, but also a witness to the pandemic (3). The vast majority were exposed to varying degrees of anxiety and panic from being isolated at home (4). In this case, what factors are related to the volunteers' working state and mental status? On the one hand, individual motivation directly has relations with the occurrence and development of voluntary behavior (5). On the other hand, the content of volunteer work also impacts volunteers in many different ways (6).

### Volunteer Motivation and Thriving at Work

Thriving at work is a mental state where energy and learning are experienced at the same time, and people with a thriving sense of work feel their own growth and motivation (7). Previous studies have found that thriving at work has a positive predictive effect on individual job satisfaction and organizational loyalty (8), and a negative predictive effect on job burnout (9). Some studies also show that individual with higher thriving at work enjoy the pleasure of work more and thus exhibit more organizational citizenship behaviors (10). At the same time, thriving at work plays an important role in individual growth and health (11). Spreitzer et al. (12) found that after controlling for variables such as depression and anxiety, subjects with high thriving at work reported better physical and mental health. Because work status is closely related to the effectiveness of psychological assistance, it is of great significance to study psychological assistance volunteers' thriving at work and mental state at work (13).

Volunteer motivation is an internal psychological process in which individuals' voluntary behaviors are guided, stimulated and maintained by their goals or objects (14). Deci and Ryan (15) proposed self-determination theory, believing that an individual has an innate and inherent tendency to meet the three psychological needs of competence, autonomy, and relationship and to achieve psychological development. Competence needs refer to the successful completion of challenging tasks and achievement of desired results. Autonomy refers to the individual feeling behavioral autonomy. Relationship needs refer to the establishment of stable and reliable contact with others, groups and organizations. If external conditions help meet the three kinds of innate psychological needs, intrinsic motivation of individuals will be enhanced, more spontaneous and active, so as to have better mental health and behavioral performance. If external conditions are not conducive to satisfaction of these three basic psychological needs, then it is not conducive to internal motivation, and the individual's work attitude and behavior performance are negatively affected to some extent (16). With regard to mechanisms producing thriving at work, based on self-determination, the socially embedded model of thriving at work has been proposed (12). Baard et al. (17) have shown that a work environment that meets individual sense of autonomy, competence and relationship needs can improve intrinsic motivation. At the same time, a large number of studies have shown that individual intrinsic motivation has a significant predictive effect on their job performance, job satisfaction, job involvement, organizational commitment, organizational citizenship behavior, innovation behavior, mental health, and subjective wellbeing (18–20). Based on previous work, the current study presents hypothesis 1, as follows:

H1: Volunteer motivation is positively related to thriving at work.

### Job Burnout, Volunteer Motivation, and Thriving at Work

Job burnout is a special type of work-related stress, which is a state of physical or emotional exhaustion that also involves a sense of reduced accomplishment and loss of personal identity (21). It includes emotional exhaustion, cynicism, and reduced personal accomplishment. Emotional exhaustion is when an individual believes that all of his or her emotional resources have been exhausted (22). Cynicism refers to the willingness of individuals to deliberately distance themselves from work and other people involved in the job (22). Reduced personal accomplishment is when an individual holds a negative opinion of himself or herself (22). Job burnout does not only harm the health of individual (23, 24), which reduces work performance of individual (25), will also increase the occurrence of bad behaviors and security accidents (26).

Internal motivation is motivation with the highest degree of autonomy, which refers to intrinsic satisfaction brought by individual activities or work itself (27). The internal motivation of work or activity is closely related to occupational stress and burnout (28). Previous studies have found that internal work motivation and external work motivation have different effects on job burnout (29). Previous evidence indicated that controlling motivation with low self-determination is in direct proportion to job burnout, while autonomous motivation with a high degree of self-determination is in inverse proportion to job burnout (30). The weakening of voluntary motivation based on one's own will is an important factor that leads to job burnout (30). Recent, under the framework of self-determined motivation theory, researchers have proposed five modes of work motivation regulation (27).

Bethencourt (31) takes the theoretical model of self-determination as its theoretical framework, with the three major psychological needs of autonomy, competence and relevance as independent variables, and individual participation as dependent variables. It detects the correlation between each other and predicts individual participation by using structural equations and regression modeling of applied data sets. The results show that there is a significant negative correlation between autonomous motivation and job burnout, while there is a significant positive correlation between controlled job motivation and job burnout. Jowett et al. (32) also indicated similar conclusions through the study of 211 professional athletes, and further proposed that the degree of individual self-determined motivation is an important factor in predicting their job burnout. Another explanation is the job demands-resources model (JD-R) proposed by Demerouti et al. **(author?)** (33). This model is a theoretical framework to systematically study the process of job burnout. In the JD-R model, one of the roles of work resources is to stimulate personal growth, learning, and development (34). At the same time, work resources correspond to the motivational process. When work demands are high, work resources are more closely related to workers' motivation (such as job involvement and job-related learning) and bring vitality to individuals (34).

Job burnout is a potential predictor of thriving at work (35). Porath et al. (7) found that thriving at work can promote sustainable development of people by influencing psychological (reduced burnout) and physiological (perceived health) aspects. Spreitzer et al. (36) pointed out that individual who were consistent with their state of thriving at work reported lower job burnout than their colleagues. Re-search by Spreitzer et al. (36) showed that thriving at work can improve job performance, reduce burnout, and improve health. Therefore, hypothesis 2 and 3 are presented as follows:

H2: Job burnout is negatively associated with thriving at work.
H3: Job burnout has a mediating effect on the relationship between volunteer motivation and thriving at work.

### Psychological Capital, Volunteer Motivation, and Thriving at Work

Psychological capital refers to a set of resources a person can use to help improve their performance on the job and their success (37). It includes optimism, hope and self-efficacy, and toughness of the four elements, respectively, representing current and future positive beliefs (optimistic), the ability to target and achieve the goal through appropriate path (hopefully), in the face of challenging tasks believe they have the ability to succeed (self-efficacy), and in the face of difficulties and adversity can stick to and hard work (toughness) (38).

When an individual participates in voluntary activities, if there is no positive psychological quality, the individual is not willing to communicate with others (39). At the same time, they tend to withdraw from difficulties and even stop their voluntary activities (40). So psychological capital plays an important role in promoting the development of voluntary behavior (39). However, in the process of the generation and development of voluntary behavior, voluntary motivation is considered to be the most important factor leading to the generation of voluntary behavior (2). That is, voluntary motivation directly affects voluntary behavior and promotes the generation and development of voluntary behavior (2). Volunteers' psychological capital quality, such as self-confidence, optimism, responsibility and hope, can stimulate individuals to produce higher voluntary motivation (41). And the improvement of volunteer motivation will further promote the generation and development of voluntary behavior (42).

According to resource conservation theory, people strive to pursue and maintain the resources they consider valuable, including autonomy, self-efficacy, self-esteem (43). Psychological capital can predict the individual's psychological development and working state (44). Individuals with a lower level of psychological capital have lower health status and lower job performance (38). Paterson et al. (45) found in their study that individual with higher level of psychological capital and more social sup-port showed more enjoyment of work and reported higher sense of work vitality. Carmeli and Spreitzer (10) also found that psychological capital has a positive effect on the working state. Specific performance is, psychological capital is higher, work is devoted, work exuberant feeling is higher. Therefore, hypothesis 4 and 5 are presented as follows.

H4: Psychological capital can positively predict thriving at work.
H5: Psychological capital has a mediating effect on the relationship between volunteer motivation and thriving at work.

**Figure 1:**
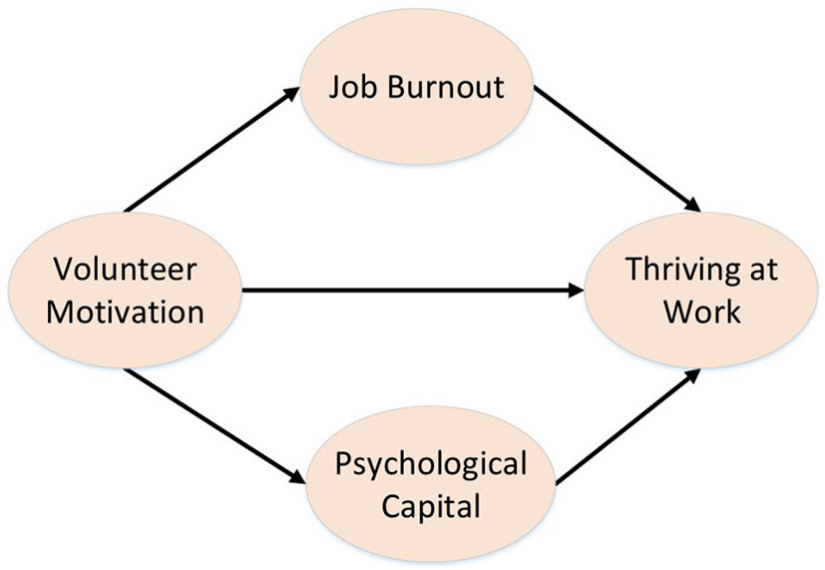
Mediation model tested.

The research model is presented as Figure 1.

## Methods

### Participants and Procedure

A cross-sectional online survey of Chinese college student between February 24 and April 19, 2020 was conducted. All participants were random recruited using the “WeChat,” a popular Chinese social media APP. WeChat has location-based online groups, and it was arranged for WeChat group (online psychological assistance college student volunteers) moderators from localities within a large urban city in Eastern China (Zhengzhou, population = 10 million) to invite their residents to participate. Interested participants were shown an online informed consent statement and, for those agreeing, a Chinese language online survey. The survey was hosted on Survey Star (www.wjx.cn), offering features to prevent automated participation by bots. Specifically, the researchers sent links of online questionnaires to WeChat groups dominated by college student volunteers who had participated in a particular volunteer activity during the COVID-19 pandemic. To further screen participants, the self-reported questionnaire included questions such as “How many times did you volunteer during this year?”

The online survey system reminded individuals to complete all items; therefore, there is no missing data. After deleting participants entering the same response consecutively across dozens of items, 349 participants remained (201 females; age: mean ± SD = 20.83 ± 2.32 years).

### Measures

In addition to surveying demographic data, the following measures were administered.

#### Volunteer Function Motivation Inventory

The Volunteer Function Motivation Inventory (VFMI) compiled by Clary et al. (46) and revised by Law et al. (47). The questionnaire is recognized as an authoritative tool to measure voluntary motivation. There are six subscales, namely, values expression, social communication, learning and understanding, career development, self-protection, and self-development. Each subscale includes 5 items. Participants rated each item according to how much it corresponded to their participation in voluntary activities with Likert 7 points (1 = strongly disagree, 7 = strongly agree). The average scores of each dimension were calculated. The higher the score, the stronger the motivation to volunteer. The Cronbach's Alpha of the total questionnaire and the six subscales were 0.917, 0.770 (value expression motivation) 0.829 (learning understanding motivation) 0.756 (social interaction motivation) 0.718 (career development motivation) 0.774 (self-protection motivation) and 0.804 (self-enhancement motivation), respectively.

#### Thriving at Work Questionnaire

The questionnaire on thriving at work was compiled and revised Porath et al. (7). The questionnaire consists of 10 items in terms of learning and vitality with Likert 7 points (1 = strongly disagree, 7 = strongly agree). The higher the score, the higher thriving at work of individual. In this study, the overall internal consistency reliability coefficient value of the questionnaire is 0.835, and that of the vitality and learning subscales are 0.780 and 0.719, respectively.

#### Maslach Burnout Inventory—General Survey

The Maslach Burnout Inventory was revised by Li and Shi (48). There are three dimensions of Emotional Exhaustion, Cynicism, and Reduced Personal Accomplishment. There are 15 questions in total. Likert ratings with Likert 7 points are used for scoring. In this study, the overall internal consistency reliability of the Burnout Inventory was 0.895. The internal consistency confidence of the three dimensions of Emotional Exhaustion and pessimism is 0.938, 0.876, and 0.894.

#### Psychological Capital Questionnaire

The PsyCap Questionnaire (PCQ)was developed by Luthans et al. (49) and translated by Li et al. (50). The scale includes four dimensions: self-efficacy, hope, resilience, and optimism. There are 6 questions in each dimension, a total of 24 questions. Likert responses are scored with Likert 6 points. The Cronbach of the scale was 0.971. The internal consistency reliability of the four dimensions of self-efficacy, hope, resilience and optimism is 0.920, 0.904, 0.924, 0.921, respectively.

### Data Analysis

SPSS software package was used to analyses the data (v. 26.0 for Windows; IBM Corporation, 2019). Mediation tests (displayed in Figure 1) were conducted using the PROCESS macro (51). Descriptive statistics and correlation analysis were used. We used the bias-corrected non-parametric percentile Bootstrap confidence interval method to analyze the mediating effect.

In the case of unrotated, Harman S One-factor Test obtained 15 factors of eigenvalue 1. The variance explained by the first factor is 30.5%, less than the upper limit of 40%. Therefore, there is no serious common method deviation problem in this study.

## Results

### Descriptive Analysis

The correlation matrix and descriptive information on the sample and the measures used in the primary analyses for this study were showed in Table 1.

**Table 1:** Description statistics and correlation analysis of each variable.

	***M***	***SD***	**1**	**2**	**3**	**4**	**5**	**6**	**7**	**8**	**9**	**10**
1. Protective	5.37	1.14	1									
2. Values	6.19	0.68	0.45^**^	1								
3. Career	4.50	1.22	0.68^**^	0.36^**^	1							
4. Social	4.85	1.08	0.68^**^	0.44^**^	0.66^**^	1						
5. Understanding	6.01	0.80	0.65^**^	0.60^**^	0.50^**^	0.55^**^	1					
6. Protective	6.01	0.80	0.68^**^	0.63^**^	0.55^**^	0.58^**^	0.86^**^	1				
7. Volunteer motivation	5.49	0.78	0.87^**^	0.66^**^	0.81^**^	0.83^**^	0.82^**^	0.85^**^	1			
8. Job burnout	2.42	0.85	−0.12^*^	−0.28^**^	−0.19^*^	−0.14^*^	−0.26^**^	−0.33^**^	−0.23^**^	1		
9. Psychological capital	4.97	0.64	0.27^**^	0.43^**^	0.19^**^	0.29^**^	0.39^**^	0.43^**^	0.39^**^	−0.61^**^	1	
10. Thriving at work	5.97	0.72	0.24^**^	0.41^**^	0.14^*^	0.27^**^	0.43^**^	0.47^**^	0.37^**^	−0.64^**^	0.61^**^	1

* *p < 0.05*,

** *p < 0.01*.

There were significant correlations between the different variables. People with higher volunteer motivation had a lower level of job burnout, and showed greater psychological capital and thriving at work.

### Mediation Model Validation Analysis

The results showed that voluntary motivation had a significant positive predictive effect on work vigor (β = 0.16, *p* <0.001). Job burnout had a significant negative predictive effect on thriving at work (β = −0.42, *p* < 0.001). Psychological capital has a significant positive predictive effect on the thriving at work (β = 0.29, *p* < 0.001; Table 2).

**Table 2:** Regression analysis of mediation models (standardized).

**Variable**	**Dependent variable**		**Dependent variable**		**Dependent variable**		**Dependent variable**	
	**thriving at work**		**job burnout**		**psychological capital**		**thriving at work**	
	**β**	***t***	**β**	***t***	**β**	***t***	**β**	***t***
Volunteer motivation	0.37	7.39^***^	−0.23	−4.30^***^	0.39	7.78^***^	0.16	3.96^***^
Job burnout							−0.42	−8.79^***^
Psychological capital							0.29	5.68^***^
*R^2^*	0.14	0.05	0.15	0.50
F	54.62^***^	18.53^***^	60.56^***^	116.46^***^

*** *p < 0.001*.

**Figure 2:**
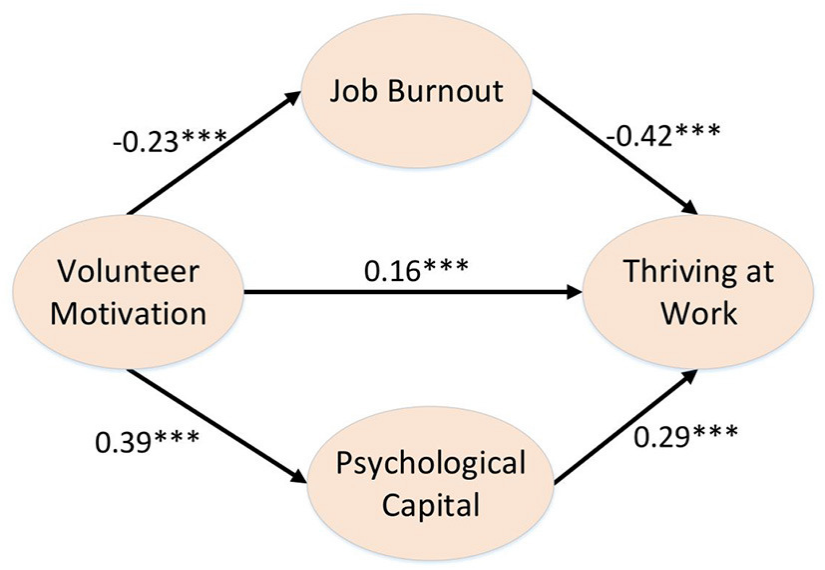
Multiple mediation model. ***p < 0.001.

The mediating effect of job burnout and psychological capital between voluntary motivation and work vigor were analyzed. The results show that the indirect effect of job burnout on voluntary motivation is 0.10. Moreover, the Bootstrap 95% confidence interval does not contain 0, indicating that job burnout has a significant mediating effect between voluntary motivation and thriving at work. The indirect effect of psychological capital on voluntary motivation was 0.11. Moreover, the Bootstrap 95% confidence interval does not contain 0, which also indicates that the mediating effect of psychological capital between voluntary motivation and work vigor is also significant (see Table 3, Figure 2).

**Table 3:** Mediation effect size analysis.

**Effect of type**	**Effect size**	**Boot SE**	**Bootstrap 95%CI**		**Relative mediation effect**
			**BootLLCI**	**BootULCI**	
Total indirect effect	0.21	0.04	0.13	0.29	55.83%
Job burnout	0.10	0.03	0.04	0.17	25.75%
Psychological capital	0.11	0.03	0.05	0.18	30.08%
Job burnout—psychological capital	−0.02	0.05	−0.11	0.09	

*Boot SE, BootLLCI, and BootULCI respectively refer to the standard error, lower, and upper limit of 95% confidence interval of the indirect effects estimated by the percentile Bootstrap method of deviation correction*.

## Discussion

### Volunteer Motivation Is Relevant to Thriving at Work

The results of this study showed that volunteer motivation can significantly positively predict thriving at work. It can be explained from three different perspectives. First, from the perspective of self-determination theory, thriving at work is related to internal motivation, and the internal work motivation of individual has a good predictive effect on their positive emotional experience, work creativity and mental health (52). Intrinsic motivation refers to the continuous interest, spirit of exploration and curiosity aroused by individual in the workplace, focusing on the effect of intrinsic motivation (53). This effect is not based on material rewards, but on certain psychological needs of the individual and the characteristics of the job itself. The main purpose of individual with high internal motivation to participate in work is not to obtain certain economic remuneration, but tends to their own interest, work activity, competency and other aspects of satisfaction (54). The cognitive state of individuals motivated by internal dynamics tends to be characterized by flexibility and persistence. And individuals are more likely to exhibit high levels of creativity and vitality (53). A study found that, other conditions being equal, individuals with high intrinsic motivation will put a lot of energy into active attempts in the process of solving problems, and their perseverance and persistence will be better (52).

The second perspective is that voluntary behavior is essentially a higher level of prosocial behavior. When individuals have prosocial motivation, they usually devote themselves to helping specific benefit groups (55). Relevant studies have shown that help-oriented prosocial motivation can produce a more lasting sense of pleasure and meaning for individuals, reduce stress, and enhance physical and mental health (56, 57).

Thirdly, according to the Action Identification Theory, when an individual takes an Action, he/she will be more concerned about the meaning of the Action (58). Individuals tend to have a high level of recognition. Work has become a carrier to express personal values and goals, enabling individuals to find meaning and sense of value in their work and making them full of vitality (59).

It can be seen that volunteer motivation has an important impact on thriving at work. Therefore, future research can deeply discuss the relationship between volunteer motivation and thriving at work from the perspectives of value expression, knowledge acquisition, functional expansion, enhanced self-esteem, self-protection, and social communication, so as to help the positive development of individual thriving at work.

### The Mediating Effect of Job Burnout and Psychological Capital

As for the mediating effect of job burnout, under the influence of volunteer motivation, individuals can reduce job burnout, and then show a stronger thriving at work. The possible reasons are: Volunteer motivation can predict job burnout (60). People with high voluntary motivation have a relatively low sense of frustration and pressure in work, so they are less likely to suffer from job burnout, and thus have a higher sense of organizational identity and thriving at work (61).

In addition, compared with job burnout, current study also found that psychological capital played a stronger mediating role in the relationship between volunteer motivation and thriving at work. Psychological capital is the comprehensive ability to meet the standard of positive organizational behavior (POB), which conforms to resource-based view (62). In other words, psychological capital is a key basic resource to manage and adjust other psychological resources to obtain satisfactory results. From the individual level, psychological capital is an important factor to promote individual growth and development and performance improvement (49). Reflected in the JD-R model, psychological capital can enhance an individual's internal motivation, make the individual feel the meaning of work, and continuously show the vitality, dedication, and concentration of work.

This study investigates the parallel mediating effect of job burnout and psychological capital, which is of great value and significance. First, the study examines the internal mechanism of thriving at work from the perspectives of emotion and cognition. This helps to provide a dual-mode intervention guide for the development of thriving at work dynamism. Secondly, this study adopts the parallel multiple mediation model. It is explained in two theoretical frameworks, namely the self-decision theory and job demands-resources model (JD-R model). This is not only conducive to the establishment of an empirical model of the influence of volunteer motivation on thriving at work, but also conducive to enterprises and organizations to take targeted measures to improve the level of individual thriving at work according to the different mechanisms of thriving at work. In addition, this study belongs to cross-sectional study. It is difficult to determine the relationship between job burnout and psychological capital. In the future, a follow-up study design could be considered to determine the relationship. To determine other possible modes of action (such as chain mediating action, etc.) of the two, thus enriching the model of thriving at work.

### Limitation and Prospect

This study examine the mediating effect of job burnout and psychological capital on the influence of volunteer motivation on thriving at work. The internal mechanism of volunteer motivation influencing thriving in volunteer work is clarified. It enriches the research on the field of thriving in volunteer work. At the same time, it also has important guiding significance for the shaping and intervention of volunteers' thriving at work. However, this study also has the following two deficiencies: (1) lack of longitudinal tracking data. It is difficult to make causal inferences. Future research should attach importance to longitudinal research design to reveal the development law of thriving at work. Then discuss how to create a positive environment conducive to the vigorous and healthy development of volunteer work. (4) In addition to the mediating effect of job burnout and psychological capital, some moderating variables, such as responsibility and competence, may also have relations with the relationship between volunteer motivation and thriving at work. Future studies could explore further mechanism of volunteer motivation on thriving at work in more complex models.

In sum, the current study indicates that volunteer motivation can significantly predict volunteers' thriving at work. The findings support a mediating relationship between job burnout and psychological capital in the relationship between volunteer motivation and thriving at work.

## Data Availability Statement

The raw data supporting the conclusions of this article will be made available by the authors, without undue reservation.

## Ethics Statement

The study was approved by the Local Ethics Committee at Tianjin Normal University, Tianjin, China. Written informed consent for participation was not required for this study in accordance with the national legislation and the institutional requirements.

## Author Contributions

JL conducted data collection, data management, cleaning, and analysis. JL and SL wrote the first draft of the paper. SL and CG substantially revised the manuscript and designed the study protocol. All authors contributed to the article and approved the submitted version.

## Funding

This study received a grant from the National Social Science Major Project of China (20ZDA079).

## Conflict of Interest

JL is a doctor student at Tianjin Normal University. CG is a paid full-time faculty member at Zhengzhou University. SL is a paid full-time faculty member at Tianjin Normal University.

## Publisher's Note

All claims expressed in this article are solely those of the authors and do not necessarily represent those of their affiliated organizations, or those of the publisher, the editors and the reviewers. Any product that may be evaluated in this article, or claim that may be made by its manufacturer, is not guaranteed or endorsed by the publisher.
